# Enlargement of the near Hock in Animals Shown to the Roller for Any Length of Time

**Published:** 1895-12

**Authors:** A. W. Clement


### REPORTS OF CASES.

### ENLARGEMENT OF THE NEAR HOCK IN ANIMALS SHOWN TO THE ROLLER FOR ANY LENGTH OF TIME.

By A. W. CLEMENT, V.S.

SOME five years ago I noticed in the show-ring that a hackney stallion did not flex the near hock as well as he did the off hock. I then noticed that most of the other stallions in the same class were affected in the same way. Then the mares were shown and I made the same observation, though the difference in the movement of the two hocks was not anything like so well marked as in the case of the stallions. I was on the outside of the ring and consequently not so well able to judge as were those inside, but I thought a good deal, and wondered if most of this beautiful and highly useful breed of animals had weak hocks, and if so, why it should constantly show itself in the near hock? The next two years I happened to be inside the ring, and I observed that in many cases the near hock was larger than the other, though perfectly smooth. Moreover, I had not yet seen any of the class lame from a practical standpoint—*i. e.*, unless all faulty action may be called lameness. A year ago I was called in consultation to express an opinion as to whether a certain hackney stallion, a prize-winner, was spavined or not. This horse had a larger hock on the near side than on the off, but the hock was smooth and the animal was not lame. I gave it as my opinion that he was not spavined.

These animals are all shown to the roller, are sent down the ring at a fast pace and then suddenly brought to a standstill, and at the same time twisted upon the near hock. Why should not the joint be more developed than its fellow? and why should not the development or enlargement be in proportion to the length of time the animal has been subjected to the treatment of being jerked on one rein continuously?

Only this summer I rejected, as unsound for road purposes, a beautiful road-mare which brought six hundred dollars under the hammer, because she was lame in the near hock. This mare had exhibited for some time beside a pony to the bridle.

She would be sent a dash past the crowd and then suddenly wheeled around for a spurt back.

A different principle applies in the case of a harness animal to that of a breeding animal. Any acquired deformity in a breeding animal can hardly be called an unsoundness. Is not the above-mentioned condition acquired and a provision of nature? At any rate, my observation is that a majority of animals shown to the side-line for any length of time will show this condition, and I should be surprised if they did not. I should like to see an autopsy, and should also like to hear the observations of others upon this subject.

